# Implementing evidence-based interventions in health care: application of the replicating effective programs framework

**DOI:** 10.1186/1748-5908-2-42

**Published:** 2007-12-09

**Authors:** Amy M Kilbourne, Mary S Neumann, Harold A Pincus, Mark S Bauer, Ronald Stall

**Affiliations:** 1 VA Ann Arbor National Serious Mental Illness Treatment Research and Evaluation Center; Ann Arbor, MI, USA; 2 Department of Psychiatry, University of Michigan; Ann Arbor, MI, USA; 3 Division of HIV/AIDS Prevention; National Center for HIV, STD, and TB Prevention; Centers for Disease Control and Prevention; Atlanta, GA, USA; 4 Department of Psychiatry, Columbia University; New York, NY, USA; 5 VA Boston Healthcare System and Harvard Medical School, Boston, MA, USA; 6 Graduate School of Public Health, University of Pittsburgh; Pittsburgh, PA, USA

## Abstract

**Background:**

We describe the use of a conceptual framework and implementation protocol to prepare effective health services interventions for implementation in community-based (*i.e*., non-academic-affiliated) settings.

**Methods:**

The framework is based on the experiences of the U.S. Centers for Disease Control and Prevention (CDC) Replicating Effective Programs (REP) project, which has been at the forefront of developing systematic and effective strategies to prepare HIV interventions for dissemination. This article describes the REP framework, and how it can be applied to implement clinical and health services interventions in community-based organizations.

**Results:**

REP consists of four phases: pre-conditions (*e.g*., identifying need, target population, and suitable intervention), pre-implementation (*e.g*., intervention packaging and community input), implementation (*e.g*., package dissemination, training, technical assistance, and evaluation), and maintenance and evolution (*e.g*., preparing the intervention for sustainability). Key components of REP, including intervention packaging, training, technical assistance, and fidelity assessment are crucial to the implementation of effective interventions in health care.

**Conclusion:**

REP is a well-suited framework for implementing health care interventions, as it specifies steps needed to maximize fidelity while allowing opportunities for flexibility (*i.e*., local customizing) to maximize transferability. Strategies that foster the sustainability of REP as a tool to implement effective health care interventions need to be developed and tested.

## Background

Closing the gap between research and practice has been a priority for many agencies, including the U.S. National Institutes of Health, Veterans Health Administration (VHA) and the Agency for Healthcare Research and Quality [1]. Despite the development of effective interventions to improve health care quality, most of these interventions have only been implemented in the academic settings in which they were developed, and few have been successfully disseminated into non-academic-affiliated (*i.e*., community-based) organizations [2]. A primary reason for this research-to-practice gap is the lack of a framework for implementing effective interventions in community-based organizations that maintains fidelity while maximizing transferability when the interventions are adopted across different settings [2-4].

An effective strategy for implementing clinical and health services (*i.e*., health care) interventions is needed. Many interventions fail to achieve the outcomes observed when tested in research settings once they are implemented in community-based organizations. This "voltage drop" in effectiveness maybe due to reduced fidelity to the intervention when disseminated outside the academic realm to community-based organizations, as well as lack of guidance in customizing interventions to community-based populations [5-8]. Moreover, stakeholders (*e.g*., health care purchasers, plans, and providers) increasingly have to choose which interventions to implement for their populations, and need guidance in implementing them in a cost-efficient manner without diminishing the intervention's effectiveness. At the same time, these stakeholders need to ensure that the intervention will be accepted and adopted across different organizations.

A number of strategies for guiding implementation efforts have been proposed [9-14], many of which primarily focus on adapting interventions by (a) determining when an organization is "ready" to adapt or adopt an intervention, and (b) working with senior leaders and frontline providers to overcome barriers to adaptation. For example, the VA's Quality Enhancement Research Initiative (QUERI) framework outlines a process for choosing, implementing, and marketing evidence-based interventions in health care organizations [12]. Simpson describes the underlying organizational characteristics that need to be considered (readiness to change, resources, and culture) when planning an implementation of evidence-based interventions [13]. Bartholomew *et al*. describe Intervention Mapping, which involves planning an implementation of a program based on information from the target population [14].

However, these frameworks do not address a fundamental issue in implementation: achieving a balance between adequate fidelity to the intervention and accommodating differences across organizations in order to maximize the effectiveness of the intervention. To date, no implementation frameworks have outlined strategies for maintaining treatment fidelity while providing opportunities to adapt interventions to fit local needs. For example, previous frameworks do not provide specific guidance in replicating interventions for use in community-based organizations, and do not specify training or technical assistance programs for facilitating implementation across different settings. Without the appropriate tools and materials available in a user-friendly format, community-based organizations are less likely to implement an evidence-based intervention; or if implemented, treatment fidelity may be suboptimal.

In this article, we describe an innovative implementation framework: Replicating Effective Programs (REP). In contrast to previously published implementation frameworks, REP provides a roadmap for implementing evidence-based interventions into community-based settings through a combination of intervention "packaging," training, technical assistance, and other strategies to maximize the chances for sustaining the interventions. REP has been empirically evaluated through a randomized controlled trial of its effectiveness in achieving intervention uptake and fidelity across different organizations [15,16]. To date, other implementation frameworks have included some but not all of the components of REP [9-14], and there have been no evaluations of implementation frameworks that combine strategies to maximize both fidelity and flexibility in implementing interventions.

### History and underlying theories of REP

REP was developed and applied by the U.S. Centers for Disease Control and Prevention (CDC) to package and disseminate HIV behavioral and treatment interventions for implementation in community-based service settings, notably AIDS service organizations [17]. CDC's REP project was initiated in 1996 to address a critical link in bridging research and practice; namely, packaging interventions so that they can be easily implemented in non-academic, community-based settings. Extensive research has focused on developing and evaluating interventions designed to change risk behaviors related to HIV transmission since shortly after the first cases of AIDS were reported in the United States [18]. The CDC's approach involved: applying rigorous criteria to identify effective interventions [19]; packaging interventions' complete scientific protocols into non-technical language [20]; supporting the implementation of the interventions through training and technical assistance [21]; and providing ongoing financial and technical support to sustain the interventions' implementation [22-24].

**Figure 1:**
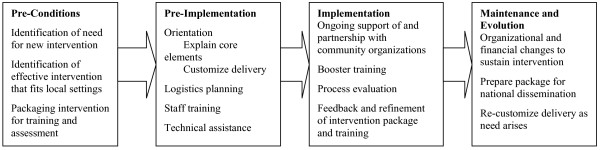
**Replicating effective programs framework for health care interventions**. This figure outlines the Replicating Effective Programs (REP) process as it can be applied to health care interventions.

The REP framework (Figure 1) builds on a systematic literature review and community input [19-24]. The concepts underlying the REP packaging process derive from action anthropology (wherein a neutral party mediates interaction and exchange between two cultures – in this case, research and practice) [25] and principles of health promotion [26]. The foundational theories for disseminating the interventions are Diffusion of Innovation, which posits that innovations pass through particular communication channels among members of a social system over time [24], and Social Learning Theory, which describes the relationship between behavior change and persons' beliefs about their ability to change and the results of the change [27].

### REP components

There are four phases to REP [22]: pre-conditions, pre-implementation, implementation, and maintenance and evolution (Figure 1). The four phases are described in detail in Table 1 as they would apply to health care interventions. When disseminating interventions, the first three phases of REP (pre-conditions, pre-implementation, and implementation) are most appropriate for the "early adopter" and "early majority" types of community-based settings based on the Rogers Diffusion of Innovation model [24]. The final phase, maintenance and evolution, is more appropriate for organizations described as the "late majority" or "laggards" based on the Rogers model, as they are more likely to participate once the intervention is already being used by other organizations and with appropriate organizational and financial incentives [24].

**Table 1:** Outline of REP process for health services-based interventions

**Phase**	**Activity**	**Process**	**Who**
**Pre-conditions**	Identify need	--Identify high-burden condition	Researchers
		--Identify barriers to implementation	
	Identify effective intervention	--Identify intervention tested in a completed, randomized controlled study	Researchers
	Identify barriers	--Organizational needs assessment, usual care	Researchers, representatives from practices, providers of community-based organizations (target population)
	Draft package	--Write package into everyday language	Intervention developers
		--Distinguish core elements, menu options	
**Pre-implementation**	Community Working Group	--Select Community Working Group (CWG)	Researchers, CWG (*i.e*., health plans, practices, providers, patients, purchasers)
		--Refine package, Core elements and menu options refined based on CWG input, adjudicated by intervention developers	
		--Refine training, technical assistance approach strategies per CWG input	
	Pilot test package	--Further refinement of package	Researchers, CWG
	Orientation	--Identify eligible organizations	Researchers, CWG, organizations participating in implementation
		--Logistics of dissemination	
		--Kick-off meeting, package dissemination	
**Implementation**	Training	--Organization staff training	Researchers and staff
	Technical assistance	--Follow-up with organizations	Technical assistance expert
	Evaluation	--Formative evaluation	Researchers
		--Model fidelity	
		--Patient outcomes	
		--Return on investment	
	Ongoing support	--Continue CWG, site visits	Researchers, CWG
	Feedback and refinement	--Analyze data, inform sustainability	CWG, Researchers
		--Refine package	
**Maintenance and evolution**	Organizational, financial changes	--CWG advises on sustainability strategies	Researchers, CWG, sites
		--Develop business case for intervention and REP process	
	National dissemination	--Reproduce package	Researchers, CWG
		--Refine business case: return on investment	
	Re-customize delivery as need arises	--Continue to refine package (*e.g*., menu options)	Researchers, CWG

### Pre-conditions

Pre-conditions for intervention adoption are: the identification of the need for a new intervention for a target population; identification of an effective intervention that fits local settings (*e.g*., mission of organization and benefits to the organization) and the intended target population (*e.g*., behavioral risks and culture); identifying implementation barriers; and drafting a user-friendly manual (i.e., "package") of the intervention.

### Identifying need

The first step of the pre-conditions phase is to identify appropriate at-risk populations and suitable evidence-based interventions.

### Identifying effective interventions

Interventions are suitable for REP if they have been rigorously evaluated (*e.g*., via randomized controlled trials or other quantitative studies) and have produced statistically significant positive effects on the health outcomes of interest.

### Ensuring intervention fits local settings

In reviewing effective interventions, researchers should be cognizant of whether the intervention has been shown to be effective in patient populations similar to those in the organizations taking part in the implementation process. Many community-based organizations serve a disproportionate number of low income, minority, and co-morbidly ill patients when compared to patients from academic settings. Another consideration is determining what the "usual care" conditions were in the effectiveness studies. Often academic practices have resources that facilitate the conduct of research studies (*e.g*., staffing, electronic medical records, space), which many community-based organizations lack.

### Identifying implementation barriers

To ensure that the intervention is feasible in local settings, researchers should meet with the staff members from participating organizations, introduce the intervention, and conduct an assessment of potential barriers to its implementation. Such meetings also foster buy-in, especially if the researchers develop a list of benefits of participation, from training opportunities to potential cost savings (*i.e*., the "business case"). During these meetings, researchers should gather information in the form of a needs assessment to benchmark usual care, including resources and functioning characteristics that may directly affect the mode by which the intervention will be implemented and the types of technical assistance that might be needed (*e.g*., staffing, patient volume, management characteristics, information technology capability). This information can inform the content of the intervention package.

### Drafting the intervention package

An intervention package is then drafted that is suitable for implementation in community-based organizations. Training and technical assistance plans are also drafted during this phase. Packaging involves the conversion of intervention protocols into non-academic language and into a user-friendly manual that can be readily disseminated [20]. The package should be drafted by the developers of the intervention, but edited by non-technical writers to ensure that the intervention package materials are free of technical jargon. Supporting documents, such as a staff training curriculum, a technical assistance guide, and promotional materials are also included. The package is refined and finalized during the pre-implementation phase of REP based on input from the pilot-testing organizations.

The REP intervention package conveys the intervention's theoretical foundation (*i.e*., core elements), components, and methods. Core elements – the critical features of the design and intent of the intervention that are thought to be responsible for the intervention's effectiveness – are essential for maintaining fidelity. Still, while core elements are standardized, the mechanisms by which they can be operationalized may vary across organizations. The package can therefore articulate menu options for adapting intervention delivery across different organizations to allow flexibility in implementation. For example, menu options for organizations with a substantial number of minorities may include culturally appropriate options for delivering the intervention, such as outreach to families or community groups. Prior evidence from the management literature suggests that articulating *a priori *the core elements and adaptation options of a new product to potential users of the product is necessary for its successful adaptation [28]. Thus, having the core elements detailed, while also providing options for implementing these core elements, is vital for optimizing both fidelity to the intervention and flexibility in its implementation.

The REP packaging process has distinct advantages over current strategies for disseminating interventions. For example, a number of behavioral health intervention toolkits (*i.e*., descriptions of the intervention components) have been published or posted on websites, such as the U.S. Substance Abuse and Mental Health Services Administration's National Health Information Center Evidence-Based Practices website [29]. However, unlike REP packages, these toolkits often lack a detailed description of the intervention's set-up procedures, underlying theories and logic flow, scripts, and other specific materials, as well as instructions for implementing these materials across different settings. In contrast, REP packages provide specific details regarding the intervention as well as operationalized options for adapting delivery of intervention core elements to local organizations in a way that does not compromise the intervention's core elements. The REP packaging process has already been widely used by the CDC to successfully implement and disseminate HIV interventions. For example, between 1996 and 2005, the REP project packaged ten interventions for use by health departments, clinics, and community-based organizations; and more packages are now being prepared. CDC and state health departments fund the implementation of REP-packaged interventions by over 500 prevention organizations nationwide [30].

### Pre-implementation

Pre-implementation involves 1) input from a Community Working Group (CWG) on developing the package, training, and technical assistance programs, 2) package pilot test, and 3) orientation and logistics in preparing the intervention for implementation.

### Community working group – develop package

To accomplish the aims of the pre-implementation phase, the intervention developer convenes a group of stakeholders from organizations serving the target population to participate in a Community Working Group (CWG). We use a comprehensive definition of stakeholders based on the Pincus multi-level 6-P framework that describes the different levels of health care: populations (i.e., communities), purchasers, plans, practices, providers, and patients (consumers) [30]. Given that these different levels play an important role in the use of REP to implement interventions, representatives from all levels should be considered for active participation in the CWG. For example, providers would be knowledgeable of the day-to-day barriers to implementing the intervention, consumers can comment on participant recruitment and burden, and health plan leaders can provide input on how to sustain the intervention beyond the REP implementation phase (*e.g*., reimbursement of intervention services). CWG participants should be persons who have influence over their peers (*i.e*., opinion leaders), yet at the same time provide input on the real-world experiences and have the clout to leverage any necessary changes to minimize barriers to intervention implementation.

The CWG members meet regularly throughout the pre-implementation phase to review the intervention materials, finalize the prototype package, advise on the staff training and technical assistance plans, and plan the logistics of the implementation. The goal of these meetings is to review and advise on the package content and develop options suitable for customizing the intervention to specific institutions (*e.g*., different options for organizing group sessions, care manager communication protocols with providers, guideline dissemination techniques). The intervention developers or researchers familiar with the science and core elements of the intervention should also participate in the CWG in order to provide expertise in the intervention. Notably, core elements and menu options are refined based on CWG input, but the final decisions to include menu options are made by the intervention developers/researchers. Whenever possible, notes should be taken at the CWG meetings to preserve insight and feedback on the package and implementation process garnered from the CWG members.

The final package should include the following components: the intervention technical manual, training curriculum, and guidance for the technical assistance. The technical manual contains a general overview (operationalized core elements, recruitment tips, and selling points for organization staff and leaders on the return on investment); intervention materials (verbatim scripts, and recommendations such as selecting an appropriate venue to conduct the intervention), as well as session workbooks and exhibits; a document describing staff members' roles, time and resource requirements, job qualifications for staff involved, and supervision guidelines; electronic files of printable materials pertinent to the intervention; and pocket cards, flowsheets, and other materials to facilitate the intervention implementation. All materials should be able to be photocopied and easy to read.

### Pilot testing the package

Once the package has been reviewed by the CWG, it can be tested for clarity and functionality within a few intervention sites. During this process, the organizations can identify five to eight patients to participate in a full intervention cycle, and study investigators should collect information on feasibility, acceptance, and any problems with the overall package, so that the package can be refined based on their input.

### Orientation and logistics

Based on input from the site visits, needs assessments, pilot test, and CWG, the prototype intervention package, training, and technical assistance plans should be finalized for distribution. The package can be distributed through orientation meetings, which can be helpful in bringing recipient organizations together as well. Prior to package distribution, a program champion should be identified at each organization. Program champions can help identify appropriate staff members who can implement the intervention and mobilize support for the intervention within their organization. Program champions, along with key staff members who will be implementing the intervention, should be asked to attend the orientation meeting.

### Implementation

The implementation phase begins upon the dissemination of the REP package to organizations, and continues with intervention training, technical assistance, and evaluation (*e.g*., fidelity and outcomes monitoring). The CWG continues to be involved in this phase to assist researchers in interpreting feedback from the evaluation process.

### Training

Training of selected staff members at participating organizations is essential in implementing and sustaining the intervention. The training should begin soon after package dissemination and should review the manuals and include role playing to showcase specific communication skills with participants. Booster training sessions should also be implemented later in the implementation process.

### Technical assistance

The technical assistance (TA) component usually occurs after training and involves regular phone calls with the organization representative within a month after training is complete. Regular calls are necessary in order for the TA experts to be proactive rather than reactive when problems regarding implementation need to be solved. The trained TA specialist takes notes during the call and documents implementation progress at each organization. He or she also advises on how to maintain fidelity (*e.g*., discerning core elements from menu options), integration with existing services, and troubleshooting the implementation process. One of the biggest challenges is ensuring that the core elements are maintained (fidelity) while its implementation is customized and adapted to local design specifications (flexibility). The TA specialist should be encouraged to discuss the tension between fidelity and adaptability with organizations, stressing that the essential core elements can be implemented using different methods while remaining consistent with the intervention's intent, and to provide positive feedback on the organization's efforts. The TA specialist should also be trained to address fidelity issues with both more- and less-experienced employees, as the former may be reluctant to change their traditional ways of patient care, while the latter may require more intensive training.

### Evaluation

Four types of evaluation strategies ought to be considered: interpretative evaluation of the intervention implementation process; measurement of intervention fidelity at the organization and patient level; patient-level outcomes; and return on investment (*e.g*., costs). Interpretative evaluation involves collection of data via qualitative interviews of providers and consumers to determine how the intervention was actually implemented, and notes from training and TA calls to determine to what extent these programs were useful for the organizations. Intervention fidelity measures should be developed to determine whether core elements were successfully implemented. For example, medical charts and staff logs can be used to count the frequency and intensity of services provided, as well as patient attendance and adherence, and staff interviews can be used to gather additional information on whether core elements were implemented correctly. Patient-level outcomes, including processes of care and clinical and functional outcomes, should be chosen based on which outcomes the intervention was designed to impact. The return on investment (*e.g*., cost-benefit, or how does the cost of implementing the intervention compare to the savings in patient care) is one of the most important evaluations to be conducted, and represents a key argument in making the business case for the intervention to stakeholders (*e.g*., health care purchasers, plans, and consumers).

## Feedback and refinement

Once the evaluation is complete, results should be vetted to the CWG for input and suggestions on how to refine the package for further dissemination. In addition, stakeholders on the CWG with knowledge and leverage over the financing of care should consider how the intervention could be sustained beyond the implementation.

### Maintenance and evolution

Maintenance and evolution involve organizational or financial changes to sustain the intervention (*e.g*., incorporation into job duties, securing funding, and training new personnel), re-customizing intervention delivery as circumstances change, and preparing the refined package, training, and TA program for national dissemination. This phase is often considered the most challenging and least studied, in part because its key component (sustaining interventions) involves concerted multilevel efforts to change the current practice and the organizational and financial incentives to make possible long-term national adoption. For example, one of the most promising strategies to sustain health services interventions is to create billable services based on the intervention's core elements. Among health care purchasers and health plans, sustainability may involve making the business case for initiating financial incentives to provide services that are crucial to the intervention. Sustaining these changes may require strategies beyond financial incentives, such as publicizing results on improved outcomes. Ultimately, good evaluation data, especially on quality performance measures and the return on investment, are essential for making the business case for these changes.

### Applying the REP process to health care settings

REP is a promising approach to implement effective clinical and health services interventions in community-based settings because many of these interventions involve complex behavioral components akin to HIV prevention interventions (*e.g*., group psychoeducation and individual self-efficacy training). There are several lessons learned from CDC's experience of REP that can be applied to implementing interventions in routine health care settings: time and resources are needed to develop prototype packages of effective interventions so that they have fidelity to the original intervention trial procedures and yet are presented in language that is friendly to community-based organizations; staff orientation and training are essential for the faithful replication of effective interventions; and when materials and training are given in ways that are accessible to community-based organizations, science-based intervention procedures are enthusiastically adopted.

Moreover, the barriers and facilitators to implementation addressed by REP experienced by AIDS service organizations are similar to those experienced in other health care settings. Table 2 presents a summary of the barriers faced by health care organizations in implementing evidence-based interventions, along with examples of how REP addresses these barriers. The Table is based on the Pincus 6-P model of barriers to implementing interventions [30]. Health care purchasers, notably Medicaid and employers, are often unaware of the array of evidence-based interventions available; nor have they been given the business case for investing in the REP implementation framework. Health plans face barriers to implementing evidence-based interventions because of the lack of financial incentives for doing so and/or because of bureaucratic complexity associated with different contractual arrangements across different provider organizations. As with many AIDS service organizations that have participated in REP, many health care settings face limited funding and bureaucratic norms that preclude many interventions from being adopted without an *a priori *implementation strategy [6].

**Table 2:** How REP addresses multilevel barriers to implementing effective treatments in health care settings

**Level**	**Barriers**	**REP components addressing barriers**
Health care purchasers	Lack of awareness of evidence-based interventions Lack of a "business case" for technology transfer models	Business case for REP process (added value of training/TA) via evaluation
Health plan	Different provider organizations fragment implementation efforts Lack of financial incentives to implement intervention	Package can be disseminated to several sites REP training Community Working Group (CWG) to include plan/purchaser and minority representatives- match model to program/state mandates
Practice organizations	Lack of time, resources to train staff in intervention Interventions not adapted to practice organization Lack of engagement if intervention is imposed on them	Manual and guidance on intervention application through TA to facilitate customization to local sites based on input from site representatives from CWG, enhance group scheduling, phone self-management sessions, and culturally appropriate options including community and family engagement
Provider	Lack of time, information Lack of training opportunities in intervention Competing priorities	REP packaging- manual on how to supplement provider services
Patient/consumer	Lack of access to customized interventions Lack of ongoing support, interest	Identification and packaging of interventions tailored to different populations; Identification of intervention core elements

In addition, many front-line providers lack the time and tools to implement interventions in everyday practice, and often feel that these interventions are being imposed on them without their input [7]. Finally, at the patient level, the HIV intervention literature has taught us that one size does not fit all, and that intervention and dissemination efforts need to be customized to particular patient populations [18,19]. This means that separate intervention packages ought to be considered for different groups if there is substantial evidence that the intervention has a differential effect on outcomes across group differences (*i.e*., moderator effects).

### Discussion: future directions

Closing the gap between research and practice is a priority, especially for community-based practices outside the academic realm. The implementation of effective interventions in community-based organizations has been impeded by the multilevel barriers across the health care system. Practical frameworks to facilitate the implementation of interventions in routine care settings are needed to facilitate a more rapid implementation of research into practice. REP is a promising tool that can be generalized to implement and disseminate clinical and health services interventions [15].

Nonetheless, REP has not been evaluated for its effectiveness beyond disseminating HIV prevention interventions, nor has it been evaluated for its effect on patient outcome or costs (return on investment). Kelly and colleagues [16] conducted the first randomized controlled trial of REP in over 70 AIDS service organizations from across the U.S. Organizations were randomized to receive the REP package, or the package, training, and TA. Those receiving the package, training, and TA were more likely to use the intervention, and intervention fidelity was greater than the package-only group. Moreover, REP was equally effective in implementing different types of HIV interventions (*e.g*., group-level and individual-level) for different populations (*e.g*., at-risk women, injection drug users, etc.) However, it was not known whether TA and training improve outcomes at the patient level above and beyond packaging alone, and whether the added time and costs of training/TA result in a more successful implementation of the intervention and improved patient outcomes.

Furthermore, a more thorough assessment of what constitutes an intervention core element is needed. Many health services interventions are limited in their ability to be replicated because they often involve multiple components that are idiosyncratic to the particular setting. Hence, a more sophisticated analysis of intervention mediators, or active ingredients, is warranted in order to develop a more precise logic flow between the intervention core element and desired outcome.

Moreover, there have been no comprehensive studies of the long-term effects of REP beyond the implementation phase (*i.e*., sustainability). For example, implementation fidelity may change in the months and years following training, and as the intervention package is disseminated to organizations that are at different stages of adoption (*e.g*., late majority, laggards, per Rogers' diffusion model). There is also little research on the degree to which this final phase could or should be tailored for different types of adopters. For example, are the late majorities and laggards more likely to respond to financial incentives, or should a technical assistance program be adapted specifically for them? Further research is needed on whether the added investment of training and technical assistance can mitigate this voltage drop in fidelity and patient-level effect, especially across the different stages of adoption. Moreover, incentives used in health services to promote long-term sustainability of an intervention, such as billing codes, performance measures, or administrative changes (*e.g*., job function changes) show promise but have not been fully studied on a large scale.

Applying the REP framework to health services interventions can potentially address these knowledge gaps, notably through studies focused on whether interventions packaged through the REP process improve patient outcomes, and which of the intervention's key components are likely to foster its sustainability beyond the implementation phase. For example, we are evaluating the effectiveness of REP in implementing a bipolar disorder care program within community-based practices using a randomized controlled trial approach (*e.g*., randomizing organizations to receive training and technical assistance versus dissemination of the bipolar disorder program package alone). Nonetheless, quasi-experimental designs with non-equivalent comparison groups are promising alternatives, especially if health care organizations are resistant to randomization, or if there are insufficient numbers of sites. Such designs can also be conducted on a larger scale, especially if patient outcomes are measurable at the population level (*i.e*., using claims data), avoiding the expense of primary data collection.

## Conclusion

Overall, REP is an valuable framework for implementing health care interventions, as it specifies steps needed to maximize fidelity to effective interventions while allowing opportunities for flexibility (*i.e*., community input) to maximize transferability. REP should be further applied to implement health services interventions and evaluated for its effectiveness in implementing different interventions in health care settings. Strategies that foster the long-term outcomes and sustainability of REP as a tool to implement effective health care interventions need to be developed and tested in health care settings.

## Competing interests

The author(s) declare that they have no competing interests.

## Authors' contributions

AMK operationalized the REP framework for use in health services, drafted the manuscript, and led revisions to the manuscript. MSN developed the REP framework and assisted with initial manuscript drafts, editing, and further manuscript revisions. HAP provided input on the application of REP to health services and assisted with manuscript preparation and editing. MSB provided input on the application of REP to bipolar disorder and the Chronic Care Model, and assisted with manuscript preparation, editing, and revisions. RS provided input on the REP framework, helped to operationalize the application of REP to health services research, and assisted with manuscript writing and final preparation.

## Disclaimer

The findings and conclusions in this report are those of the authors and do not necessarily represent the views of the Centers for Disease Control and Prevention or the Department of Veterans Affairs.
